# Decolonisation and quality of care

**DOI:** 10.1136/bmj-2022-071585

**Published:** 2023-03-13

**Authors:** Bernice Yanful, Meghan Bruce Kumar, Ezequiel García-Elorrio, Chris Atim, Sanam Roder-DeWan

**Affiliations:** 1 Dalla Lana School of Public Health, University of Toronto, Toronto, Canada; 2 London School of Hygiene and Tropical Medicine, London, UK; 3 KEMRI-Wellcome Trust Programme, Nairobi, Kenya; 4 Institute for Clinical Effectiveness and Health Policy, Buenos Aires, Argentina; 5 Results for Development (R4D), Accra, Ghana; 6 World Bank Group, Washington, DC, USA; 7 Dartmouth Medical School, Hanover, NH, USA

## Abstract

Delivering high quality healthcare for all requires recognising the legacies of colonialism in driving power asymmetries and producing inequitable health outcomes both within and between countries say **Bernice Yanful and colleagues**

Colonialism continues to shape local health systems and access to high quality care. The 2013-16 Ebola crisis in west Africa, for example, has roots in a colonial history of extractive mining industries, which continue to divert critical financial resources from the region leaving health systems underfunded.^1^ ^2^ Consequently, when Ebola broke out, patients’ quality of care was undermined by vulnerabilities in their local health systems, including medication and workforce shortages. This was coupled with a poorly coordinated global response that accepted lower standards of care for those living in the global south.^1^ ^2^ Such inequities can be traced back to ideologies of oppression and exploitation, which assign different values to human life based on factors such as skin colour and place of origin.

The field of quality improvement in healthcare has tended to favour interventions that focus on individuals, such as clinical training, yet these inadequately engage with the systemic roots of health inequities.^3^ This approach mirrors clinical diagnosis and treatment patterns in specialties of western medicine that focus on specific diseases or organ systems over the person as a whole. A decolonial approach to high quality care for all requires reflexivity and action at the level of health systems.

## Decolonise standards, measurements, and quality improvement

We must start by challenging the implicit acceptance of lower standards of quality and higher clinical risk in healthcare populations with less political power. For example, current European and North American guidelines on perinatal care are based on evidence that antenatal transport is safer than intrapartum or postpartum transfers,^4^ ^5^ and these systems are therefore designed to have nearly all women deliver in hospital or close to emergency services to decrease risks. In contrast, global guidelines,^6^ applied almost exclusively to low income post-colonial countries, allow for a “basic” level of childbirth care without surgical services or blood transfusion, which in emergencies rely on referral to higher levels of care. Transportation of a woman with an intrapartum complication or a sick newborn is challenging even with good roads and advanced life support ambulances staffed by skilled providers; in many settings such transport happens over long distances, poor roads, and without an accompanying provider or clinical care, making transfer dangerous. Highlighting such double standards may push health systems to change course, develop innovative solutions to facilitate access to comprehensive services before labour begins, and help achieve more equitable and effective systems.

Measuring quality is also critical for improving its delivery. Perceptions of quality are shaped by cultural and societal values, which makes measuring them context specific. For example, western medicine has its roots in individualistic cultures that value privacy, and thus measures of privacy are common in global quality frameworks. However, privacy may not be as highly valued in collectivist cultures.^7^ Although using appropriate global standards of technical quality can provide opportunities for comparison between settings, the validity of measures of user experience is proportional to the diversity of voices included. Such measures should be validated and tested locally before they are used. Efforts to impose measurement frameworks created by the global north perpetuate colonial relations of power and dominance^2^ and risk contextual irrelevance.

A systems-led decolonial approach responds to local needs and priorities across the health system. Yet, too often ideas for quality improvement originate from “best practice” in the global north. We need greater south-north learning that prioritises mutual learning and knowledge transfer, and builds capacity for locally responsive interventions^8^ that strengthen the delivery of care while honouring the unique contexts of patients, families, and communities. Decolonising education for healthcare professionals, which is often linked to colonial standards and institutions, is a critical step in improving quality. Curriculum changes may include redressing the lack of darker skin tones in clinical learning resources,^9^ teaching the history of colonial medicine, exploring the role of colonialism in creating social divisions that play out in patient care and respect, and teaching skills to identify and distinguish various knowledge systems and therapeutic models, including one’s own.

A genuinely decolonial approach should focus on identifying and addressing the systemic imbalances of power within and between societies that lead to inequities. By recognising and centring the systemic roots of health and illness, we can move towards ensuring that all individuals, communities, and populations receive care that “increase[s] the likelihood of desired health outcomes.”^5^ This approach requires challenging mainstream conceptions of what constitutes high quality of care and proposing alternative paths to achieve it.

## Notes

Provenance and peer review: Commissioned; externally peer reviewed.

This article is part of a collection proposed by the World Health Organization and the World Bank and commissioned by *The BMJ*. *The BMJ* peer reviewed, edited, and made the decision to publish these articles. Article handling fees are funded by the Bill and Melinda Gates Foundation. Jennifer Rasanathan, Juan Franco, and Emma Veitch edited this collection for *The BMJ*. Regina Kamoga was the patient editor.
